# Effect of Different Post Materials and Adaptability on Fracture Resistance and Fracture Mode in Human Endodontically Treated Teeth

**DOI:** 10.1155/2022/9170081

**Published:** 2022-08-04

**Authors:** Anutara Bhaktikamala, Wareeratn Chengprapakorn, Pravej Serichetaphongse

**Affiliations:** ^1^Private Practice, Bangkok, Thailand; ^2^Esthetic Restorative and Implant Dentistry International Program, Faculty of Dentistry, Chulalongkorn University, Bangkok, Thailand; ^3^Prosthodontics Department, Faculty of Dentistry, Chulalongkorn University, Bangkok, Thailand

## Abstract

**Objective:**

To investigate the effect of different post materials and adaptability on fracture resistance and fracture mode of endodontically treated teeth.

**Materials and Methods:**

Sixty extracted human mandibular premolars were selected and divided into 6 groups (*n* = 10) according to the restorative method after endodontic treatment: no ferrule presented and restored without fiber post (Group C), 2.0 mm ferrule presented and restored without fiber post (Group CF), restored with D.T. Light-Post (Group PDT), restored with anatomically customized D.T. Light-Post, relined with resin composite (Group ADT), restored with Hi-Rem prosthetic post (Group PHR), and restored with anatomically customized Hi-Rem prosthetic post, relined with resin composite (Group AHR). After restoring with core build-up materials, all specimens were loaded at 45° in a universal testing machine until failure. Visual inspection of all specimens for fracture modes was performed. The data were analyzed using one-way ANOVA, and the fracture mode was reviewed using the chi-square test.

**Results:**

Anatomically customized groups presented statistically significant higher fracture resistance than prefabricated groups and group C (*P* < 0.05). Without post, group CF displayed significantly higher fracture resistance than group C (*P* < 0.05). Group C, CF, PDT, and PHR showed some specimens with unfavorable fractures.

**Conclusions:**

Anatomically customized posts presented highest fracture resistance among all groups. There was no significant difference in fracture mode across all groups.

## 1. Introduction

Many studies reported that the most common failure in endodontically treated tooth (ETT) was not from endodontic treatment but rather from the prosthetic aspect [1, 2]. One of the key factors to long-term success of the restoration is bonding procedure [3, 4]. In ETT with extensive loss of tooth structure, post and core are usually needed to provide resistance and retention for the definitive restorations [5–8]. Different materials, post systems, and fabrication techniques, such as metal alloy cast post, fiber reinforced post, and CAD/CAM fabricated post, have been proposed to restore lost tooth structures [9–13]. Traditionally, metal alloy cast post has been a widely accepted procedure due to its intimate adaptation to post space and high fracture resistance [14]. However, some of the disadvantages of this metal alloy cast such as the stiffness, stress distribution pattern, color incompatibility, and catastrophic fracture outcomes are among the concerns [15, 16].

Fiber-reinforced resin composite (FRC) post has become an alternative treatment option for ETT from its improved esthetics result and ease of manipulation. Since FRC post has similar modulus of elasticity to that of dentin, it provided better stress distribution to the remaining radicular dentin [11, 17–19]. Nevertheless, to achieve better flexural strength of the post, zirconia posts have been also introduced [20]. However, stiff zirconia post lacks plastic behavior and possibly contributes to catastrophic failure [21, 22]. Meanwhile, zirconia-reinforced fiber post exhibits more similar elastic modulus to dentin, promoting high fracture resistance and preventing catastrophic failure [23].

D. T. Light-Post (RTD Dental, Saint-Egrève, France) is one of the widely used FRC systems with high fatigue resistance and successful outcome [24]. Although restoring with fiber posts show mostly noncatastrophic or reparable fracture outcomes, the remaining fiber post in root canal is very difficult to remove when failure occurs. Hi-Rem post (Overfibers, Bologna, Italy), which contains soft polymer macrofiber in the middle of the zirconia glass fiber post, was introduced and reported to have comparable bond strength to D. T. Light-Post and ease for removal when failure occurred [25]. However, studies regarding fracture resistance and fracture mode of this post system are scarce. On the contrary to metal cast post, prefabricated fiber post usually does not adapt well to the conformity of the post space, especially in excessive loss of tooth structure and flared root canals in maxillary central incisors or mandibular premolars [26–28]. This poor adaptation of the fiber post results in thick and uneven cement layer which may increase the risk of air entrapment and flaws in the cement layer. Hence, higher polymerization shrinkage stress and chances of debonding at the dentin-cement and post cement interfaces may occur [29–32].

Several techniques have been proposed to improve the adaptation of the fiber post to the post space, including indirect anatomical fiber post, CAD/CAM post, and anatomically customized fiber post [30, 33–36]. Despite various proposed materials, post systems, and fabrication techniques, there is no consensus concerning which option is the most appropriate approach. Also, there were only few studies comparing restored ETT with different fiber post materials and fabrication techniques. Therefore, the aim of this study was to analyze the effect of different post materials and adaptability on fracture resistance and fracture mode of ETT. The null hypotheses were as follows: (1) there is no difference in the fracture resistance of ETT using different post materials and adaptability and (2) there is no difference in the fracture mode of ETT using different post materials and adaptability.

## 2. Materials and Methods

This study protocol has been approved by the ethical committee. The total sample size was determined using the G*∗*Power program and data from prior research using a similar experimental design [13]. The suggested sample size was at least 8 specimens in each group. Human mandibular single-root premolars extracted less than 6 months were selected. All teeth were inspected with PeriOptix Loupe (DenMat, California, USA) under 3.5× magnification and transillumination examination to exclude cracks, caries, open apex, and restorations. Total of 60 teeth with similar buccolingual root dimension of 7.1 ± 0.5 mm and length from buccal cementoenamel junction (CEJ) to apex of 14.2 ± 0.6 mm were selected after measuring by digital caliper (Mitutoyo Corporation, Kanagawa, Japan) and periapical films. Following that, 60 teeth with similar dimensions were numbered and randomized by a number randomizer (Research Randomizer Version 4.0, Urbaniak, G. C., and Plous, S 2013) into 6 groups with 10 specimens each. Subsequently, the teeth were cleaned and stored in distilled water.

Radiographs with K-file no. 15 inserted into the root canals were taken to check patency and root canal conditions. The coronal part of the tooth was sectioned 2 mm above buccal CEJ perpendicular to the tooth axis. Post systems used in this study were D. T. Light-Post (Quartz Fiber, Double-tapered, *Ø* = 1.5 mm at cervical, 0.9 mm at apical, Batch #463632007), and Hi-Rem Prosthetic Post (Zirconia Glass Fiber, cylindrical-conical, *Ø* = 1.6 mm at cervical, 0.8 mm at apical, Batch #I240359). Elastic modulus of materials mentioned in this study is shown in Table 1.

### 2.1. Specimen Preparation

No. 10 K-file was used to measure the working length. The roots were endodontically treated at the working length of 1 mm from apical foramen using ProTaper Next rotary system (Dentsply Sirona, North Carolina, USA). Root canal treatment were prepared with 21 mm·M-Wire NiTi rotary files ProTaper Next X1, X2, and X3 (Dentsply Sirona, North Carolina, USA). They were driven by X-Smart endodontic rotary motor (Dentsply Sirona, North Carolina, USA) with recommended 3 Ncm torque at a maximum speed of 300 rpm. The last finishing instrument was ProTaper Next X3 (0.30 mm tip with 7% taper). During each instrumentation, the root canals were irrigated with 2.5% sodium hypochlorite (NaOCl). When instrumentation of the root canal was completed, 17% ethylenediaminetetraacetic acid (EDTA) solution was applied for 1 minute. Canals were flushed again with 2.5% NaOCl and of normal saline solution for 1 minute to remove remaining of EDTA and dried with paper points. The prepared root canals were obturated with gutta percha (Dentsply Maillefer, Ballaigues, Switzerland) and AH Plus Jet noneugenol sealer (Dentsply Maillefer, Ballaigues, Switzerland). Afterward, the access opening was restored with Cavit temporary filling material (3 M ESPE, Minnesota, USA) and stored in 100% humidity at 37°C for 24 hours.

Gates-Glidden drills (Dentsply Maillefer, Ballaigues, Switzerland) were used to remove gutta percha maintaining 5 mm of gutta percha for apical seal and confirmed by periapical radiographs. Peeso reamer drills (Dentsply Maillefer, Ballaigues, Switzerland) were used to flare root canals to the standardized radicular dentin wall of 2 mm at cervical. Afterward, taper diamond bur at slow speed was used to create controlled similar smear layer on the post space surfaces of each specimen. The post spaces were prepared to leave 5 mm of gutta percha from root apex and surveyed to be parallel with the root axis. Outer surface of the roots was coated with thin layer of polyvinyl siloxane to replicate the periodontal ligament at 2 mm below the buccal CEJ. Polyvinyl chloride (PVC) rings were used as molds for specimens. The specimens were placed in acrylic resin using a surveyor to position the roots perpendicular to horizontal axis. All specimens were then submerged in water at room temperature to prevent overheating from polymerization.

The specimens were allocated into six groups with 10 specimens in each group. There was no significantly different mean root dimension between groups (*P* > 0.05).

Group C: specimens were restored without fiber posts. The post space was filled with Built-it FR fiber reinforced core material (Kerr, California, USA).

Group CF: specimens had 2 mm ferrule and were restored without fiber posts. The post space was filled with Built-it FR fiber reinforced core material.

Group PDT: specimens were restored using size ^#^1 prefabricated D. T. Light-Post.

Group ADT: specimens were restored using size ^#^1 prefabricated D. T. Light-Post and relined with Filtek Z350XT resin composite (3 M ESPE, Minnesota, United States).

Group PHR: specimens were restored using size ^#^2 prefabricated Hi-Rem prosthetic post.

**Figure 1:**
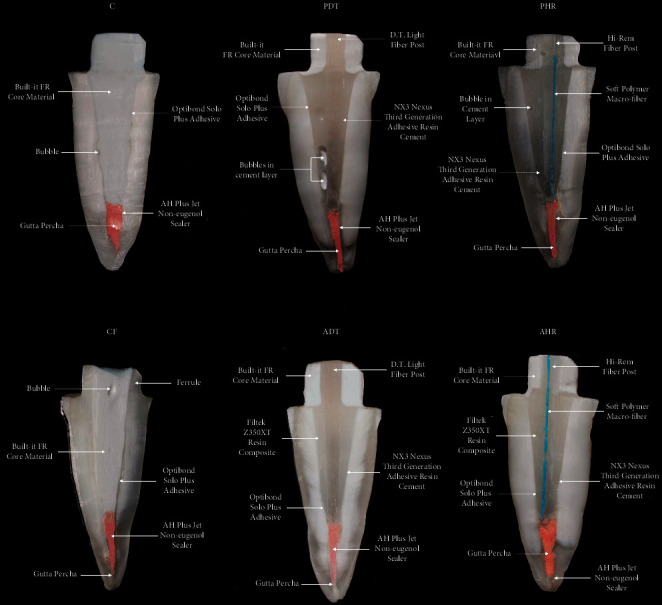
Sectioned specimens presented components in each experimental group.

Group AHR: specimens were restored using size ^#^2 prefabricated Hi-Rem prosthetic post and relined with Filtek Z350XT resin composite. Components in each specimen groups were presented in Figure 1.

All teeth underwent the same adhesive treatment using total-etch dental adhesive. Dentin was etched for 15 seconds with 37% phosphoric acid and rinsed thoroughly for 15 seconds before gently air-dried. Paper point was used to remove moisture. OptiBond Solo Plus adhesive (Kerr, California, USA) was applied to both post space and post surface with an applicator tip for 15 seconds, using light brushing motion and gentle air-blow for 10 seconds. Excess adhesive was removed with dry applicator brush to avoid pooling of adhesive before light-curing for 20 seconds.

For anatomically customized groups, post spaces were lubricated with KY gel water-based lubricant (Reckitt, Berkshire, England). Adhesive was applied on the post surface with the same protocol as in prefabricated group and light-cured for 20 seconds. Filtek Z350XT resin composite were applied onto fiber posts and inserted into post spaces. Subsequently, the fiber posts adapted with resin composite were light-cured for 20 seconds within the post spaces. The posts were removed from canal for further 40 seconds of light curing to complete the polymerization process. This procedure was repeated until the posts achieved the conformed shape of post spaces and then cleaned with water and alcohol.

NX3 Nexus Third Generation dual-cure adhesive resin cement (Kerr, California, USA) was used for bonding in all post groups. The posts were initially inserted into canals and held in seated position with finger pressure. Excess cements were removed with cotton pellets and light cured. After the post cementation, standardized custom core formers with a diameter of 3.6 mm made from Memosil 2 translucent polyvinylsiloxane (Kulzer GmbH, Hanau, Germany) were used to create core with Built-it FR fiber reinforced core material (Kerr, California, USA). The specimens were kept humid for 7 days at 37°C prior to fracture testing.

### 2.2. Fracture Resistance Test

**Figure 2:**
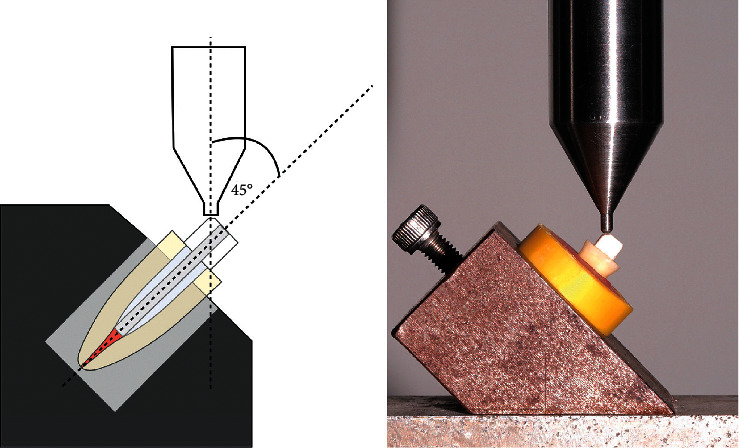
Illustration of the fracture resistance test set up: the specimen holder secured the position of each sample at 45° off-axis.

Each specimen was positioned on the mounting device and aligned at 45° angle with respect to the long axis of the root in Lloyd LR 10 K universal testing machine (Lloyd Instruments Ltd., West Sussex, UK), using a cylindrical-shaped device with a round tip (2.0 mm in diameter) at a crosshead speed of 0.5 mm/minute until fracture. The load was applied at the linguo-occlusal surface of the coronal portion of the cores and measured in Newtons (N) as shown in Figure 2. Fracture resistance was defined as the point at which the loading force reached a maximum value before fracturing the root or core, bending, or debonding the post.

### 2.3. Fracture Mode

After fracture resistance test, all specimens were visually inspected under 3.5× magnification and transillumination to determine the type, location, and direction of the fracture failure. The fracture modes were categorized based on the restorability of the tooth. The specimens with fracture in the cervical third of the roots were classified as favorable or restorable mode, whereas specimens with fracture in the middle and apical third of the roots were classified as unfavorable or irreparable mode.

### 2.4. Statistical Analysis

Data distribution was determined by using the Shapiro–Wilk normality test. The fracture resistance data was analyzed using One-Way ANOVA. The fracture mode data was evaluated using the chi-square test. Statistical analysis was performed by using the SPSS 20.0 software (SPSS Inc., Illinois, USA).

## 3. Results

From Shapiro–Wilk normality test, this in vitro study presented normal distribution. The mean fracture resistance and standard deviation are presented in Table 2. According to the Tukey HSD test, group AHR showed the highest fracture resistance compared to the other groups. While group C recorded the lowest fracture resistance, the fracture resistance of anatomically customized groups in both post systems were significantly higher than prefabricated groups and group C (*P* < 0.05). Group CF presented with significant higher fracture resistance than group C (*P*=0.027). However, group CF showed no statistically significant difference to prefabricated and anatomically customized groups (*P* > 0.05). No significant different fracture resistance was found between group AHR and group ADT (*P*=0.998). Moreover, no statistically significant difference was found among prefabricated groups and group C (*P* < 0.05).

**Figure 3:**
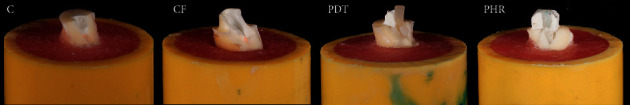
Samples with unfavorable fracture mode found in group C, CF, PDT, and PHR.

The fracture mode was visually inspected and analyzed as shown in Table 3. Almost all fractures occurred at the cervical third area of the roots, which represented the favorable outcome (91.7%). One sample (10%) in each prefabricated groups and group CF was reported with unfavorable fracture mode. While two samples (20%) in group C exhibited unfavorable fracture mode as shown in Figure 3. According to the chi-square test, there was no statistically significant difference in the failure mode in all groups (*P*=0.592).

## 4. Discussion

Not only effective endodontic treatment is required to ensure a successful and long-term outcome of ETT, but reliable prosthetic treatment is also critical, as it has been demonstrated to be the most prevalent cause of failure in numerous studies [1, 2]. Restoration of ETT, particularly with flared root canals, remains a challenging procedure. This is due to the mismatch between the broad diameter of the post space and the size of the prefabricated fiber post, which leads to poor retention and thick cement layer. The procedure could be compromised since the residual tooth structure may be insufficient to withstand masticatory forces, making the teeth prone to fracture [11–13]. In accordance with the results of this study, the advantages of adapting resin composite to the post were established as there was statistically significant difference in fracture resistance related to the fabrication technique (*P* < 0.05).

Fracture resistance of specimens ranged from 585.9 ± 28.1°N in group C to 688.4 ± 56.4°N in group AHR. Groups ADT and AHR displayed statistically significant higher mean fracture resistance compared to prefabricated groups and group C with mean load of 679.8 ± 57.8°N and 688.4 ± 56.4°N, respectively (*P* < 0.05). These findings could be explained by intimate contact between anatomically customized post and post space, contributing to increased frictional retention and a thin, homogeneous cement layer. Well-adapted post minimized post and core movement under occlusal loading. This resulted in better stress distribution throughout post, adhesive, and dentin which also supported by previous studies [32, 35].

Likewise, multiple studies established a beneficial direct effect of anatomically customized post on ETT fracture resistance and bond strength [13, 26, 34]. The close and uniform contact of post to residual root also contributed to higher sustained seating pressure in cementation process which led to better adhesive interfaces quality [3]. The mentioned advantages in adhesive quality and bond strength resulted in less risk of restoration failure since it showed to be where the restoration failure frequently occurred [26]. Moreover, the finite element analysis indicated that the customized fiber posts displayed lower stress concentration in dentin and post compared to prefabricated fiber post [32]. Three-year follow-up case report studies supported that this fabrication technique could be used as a successful alternative to metal alloy cast post to increase the fracture resistance and bond strength [27, 36].

On the contrary, a study reported that when restoring with fiber posts and direct resin composite crown on tooth with absence of ferrule showed similar fracture resistance with no effect from different fit and form-congruence of the posts to post space [18]. Nonetheless, there was only 0.3 mm difference between post diameter and post space in no form-congruence group in the mentioned research. Regarding bond strength, a previous study showed that there was no significant different in bond strength among different diameters of posts and sizes of post space [28].

CAD/CAM post had been introduced to improve adaptation of post to residual root, similar to relining fiber post with resin composite. They demonstrated comparable high fracture resistance and push-out bond strength. Regardless, the CAD/CAM technology and materials used were not as economical friendly as the latter fabrication technique, requiring costly milling machine and maintenance. In addition, less chair time and post fabrication process could be carried out by relining fiber post with resin composite [4, 9, 13]. Due to the reasons previously mentioned, this present study excluded the use of CAD/CAM post.

The statistically significant lower fracture resistance of the prefabricated fiber post (*P* < 0.05) in this study could be caused by the mismatch of post size to post space in both post system. Poor adaptation of posts to the post space resulted in thick and inconsistent cement layer, increasing the risk of defects in adhesive and cement layer as found in Figure 1. Recent research showed that when ETT was restored using prefabricated fiber posts with a thick layer of resin cement, the area of air bubbles in the cement layer was greater than when customized fiber posts relining with resin composite were used [30]. Under occlusal loading, these flaws could induce stress and initiate crack propagation which lead to debonding, dislodgement, and fracture of restoration [35]. In addition, thick cement layer also increased polymerization shrinkage stress, as it proportionally relates to the volume of resin cement. Occasionally, the bond strength between luting cement and radicular dentin was insufficient to withstand polymerization shrinkage stress in the presence of thick cement [4]. This also aligned with studies that one of the most common failure of ETT restored with prefabricated fiber posts was debonding resulted from technical sensitivity of bonding procedure in post space [26,31]. Likewise, a study stated that the junction between the resin cement and the root dentin of prefabricated fiber post was where all failures occurred [26].

Different post materials and systems did not influence the fracture resistance in both fabrication techniques (*P* > 0.05) in this study. This might be explained by the similarity of shape, size, and mechanical properties among the post systems. Frequently, the failure of post and core restoration was inevitable. Fiber post removal of failed restoration in ETT was challenging. The Hi-Rem fiber post was reported in the previous research to have similar bond strength while required less removal time when compared to D. T. Light-Post [25]. According to the data of this present study, it occurred that both post systems presented similar fracture resistance (*P* > 0.05).

In the current study, the assumption that restoring ETT with different post materials and adaptability would result in different failure modes could not be sustained based on the available evidence (*P* > 0.05). Only two specimens from group C and one specimen in group PDT, PHR, and CF exhibited unfavorable fracture mode as shown in Figure 3. This resulted from similarity of post systems and materials used as previously stated. Moreover, using resin cement, resin composite, core build-up material, and posts with comparable modulus of elasticity to dentin created mechanically homogeneous restoration as reported in previous studies [16, 29]. The similarity of mechanical properties permitted the loading stress to distribute evenly, resulting in more favorable failure mode. The better stress distribution pattern and reparable failure mode of fiber post were also supported by various studies [5, 19]. Likewise, a finite element analysis study showed that fiber post displayed better uniformity in stress distribution compared to stiffer material such as gold cast post [16].

The remaining tooth structure is shown to be a crucial factor to the prognosis and longevity of ETT. The ETT with considerable loss of tooth structure was susceptible to withstand the occlusal loading forces [6]. From the result of the present study, group CF with 2.0 mm ferrule showed significantly higher fracture resistance compared to group C with no ferrule (*P* < 0.05). In accordance with a study, the fracture resistance of ETT was directly related to remaining dentin wall [8]. This was also supported by another study, stating that the remaining dentin with such amount of ferrule and its uniformity had a significant effect on fracture resistance and allowed better occlusal load dissipation [7]. Contrary to established recommendations for post insertion regarding ferrule, the authors purposely excluded ferrule from all but group CF. The first objective was to minimize influencing factors. Second, it was intended to examine the possibility of restoration in the case of significant tooth structural loss resulted in no ferrule. Additionally, it aimed to provide more conclusive evidence for the requirement of the ferrule.

Nevertheless, the relation of remaining radicular dentin to fracture resistance was still a controversy. Various studies stated that less remaining radicular dentin in flared root canal from extensive carious lesion, trauma, or iatrogenic cause was more prone to root fracture [13, 17]. While a study showed that the remaining radicular dentin thickness of 1 or 2 mm was not a significant factor regarding fracture resistance [12]. However, from the mentioned study, all samples in the group with remaining radicular dentin of 1 mm and metal alloy cast posts resulted in irreparable failure. Similarly, with 2 mm of remaining radicular dentin at cervical in the present study, there was no statistically significant difference in fracture mode among restoration groups (*P* > 0.05).

This study is an in vitro study that did not replicate actual intraoral environment. Temperature and humidity were not similar to the real clinical situation. Load was applied only in a single direction. Further investigations might consider more samples, others fabrication techniques, different restorative materials, different luting systems, artificial saliva bath, thermocycling, and fatigue resistance testing.

## 5. Conclusions

Within the limitations of this current study, it was concluded that ETT restored with anatomically customized post had higher fracture resistance to ETT restored with only prefabricated post and without post. The fracture mode was not influenced by post materials and adaptability. Ferrule was essential for fracture resistance in restoring endodontically treated tooth with flared root canal.

## Figures and Tables

**Table 1:** Elastic modulus of mentioned materials.

Material	Modulus of elasticity (GPa)	Number of times compared to dentin	References
Dentin	18.6	1	[16,29]
DT Light-post® illusion® X-RO®	15	0.81	[37]
Hi-rem prosthetic post	60	3.23	[38]
Gold	90	4.19	[21]
Zirconia post	200	10.75	[21]
Metal post	208	11.18	[39]
NX3 dual-cure resin cement	9.5	0.51	[40]
Filtek™ Z350XT resin composite	11.3	0.61	[41]
Built-it FR core material	15.5	0.83	[42]

**Table 2:** Mean and standard deviation of fracture resistance values (*N*) of study groups.

Study groups	*N*	Mean (*N*)/SD	Min, max
C	10	585.9 ± 28.1^a^	554.9, 628.1
CF	10	649.5 ± 44.2^bc^	593.2, 708.1
PDT	10	616.2 ± 39.9^ab^	561.3, 677.5
ADT	10	679.8 ± 57.8^c^	612.1, 778.5
PHR	10	607.2 ± 32.4^ab^	572.8, 653.2
AHR	10	688.4 ± 56.4^c^	608.4, 751.3

Same superscript indicated no statistically significant difference, analyzed by one-way ANOVA and tukey HSD post-hoc test (*P* > 0.05).

**Table 3:** Number and percentage of failure modes observed in each experimental group.

Study groups	Number (percentage within group)	
	Favorable	Nonfavorable
C	8 (80)	2 (20)
CF	9 (90)	1 (10)
PDT	9 (90)	1 (10)
ADT	10 (100)	0 (0)
PHR	9 (90)	1 (10)
AHR	10 (100)	0 (0)

Pearson chi-square test showed no statistically significant different in failure modes (*P*=0.592).

## Data Availability

The data that support the findings of this study are available from the corresponding author upon reasonable request.

## Additional Points

Clinical significance: anatomically customized posts exhibit high fracture resistance when used to restore endodontically treated tooth with extensive loss of tooth structure.
